# The Dynamic Changes of Transcription Factors During the Development Processes of Human Biparental and Uniparental Embryos

**DOI:** 10.3389/fcell.2021.709498

**Published:** 2021-09-17

**Authors:** Chenxi Zhang, Conghui Li, Ling Yang, Lizhi Leng, Dragomirka Jovic, Jun Wang, Fang Fang, Guibo Li, Depeng Zhao, Xuemei Li, Lin Lin, Yonglun Luo, Lars Bolund, Jinrong Huang, Ge Lin, Fengping Xu

**Affiliations:** ^1^College of Life Sciences, University of Chinese Academy of Sciences, Beijing, China; ^2^BGI-Shenzhen, Shenzhen, China; ^3^Lars Bolund Institute of Regenerative Medicine, BGI-Qingdao, BGI-Shenzhen, Qingdao, China; ^4^Qingdao-Europe Advanced Institute for Life Sciences, BGI-Shenzhen, Qingdao, China; ^5^China National GeneBank, BGI-Shenzhen, Shenzhen, China; ^6^School of Basic Medical Science, Institute of Reproductive and Stem Cell Engineering, Central South University, Changsha, China; ^7^Key Laboratory of Reproductive and Stem Cells Engineering, Ministry of Health, Changsha, China; ^8^Reproductive and Genetic Hospital of CITIC-Xiangya, Changsha, China; ^9^Department of Reproductive Medicine, Affiliated Shenzhen Maternity and Child Healthcare Hospital, Southern Medical University, Shenzhen, China; ^10^Department of Biomedicine, Aarhus University, Aarhus, Denmark; ^11^Steno Diabetes Center Aarhus, Aarhus University Hospital, Aarhus, Denmark; ^12^Department of Biology, University of Copenhagen, Copenhagen, Denmark; ^13^BGI Cell, BGI-Shenzhen, Shenzhen, China

**Keywords:** uniparental embryos, single-cell RNA sequencing, transcription factors, gene expression, embryo development

## Abstract

Previous studies have revealed that transcription factors (TFs) play important roles in biparental (BI) early human embryogenesis. However, the contribution of TFs during early uniparental embryo development is still largely unknown. Here we systematically studied the expression profiles of transcription factors in early embryonic development and revealed the dynamic changes of TFs in human biparental and uniparental embryogenesis by single-cell RNA sequencing (scRNA-seq). In general, the TF expression model of uniparental embryos showed a high degree of conformity with biparental embryos. The detailed network analysis of three different types of embryos identified that 10 out of 17 hub TFs were shared or specifically owned, such as ZNF480, ZNF581, PHB, and POU5F1, were four shared TFs, ZFN534, GTF3A, ZNF771, TEAD4, and LIN28A, were androgenic (AG) specific TFs, and ZFP42 was the only one parthenogenetic (PG) specific TF. All the four shared TFs were validated using human embryonic stem cell (hESC) differentiation experiments; most of their target genes are responsible for stem cell maintenance and differentiation. We also found that Zf-C2H2, HMG, and MYB were three dominant transcription factor families that appeared in early embryogenesis. Altogether, our work provides a comprehensive regulatory framework and better understanding of TF function in human biparental and uniparental embryogenesis.

## Introduction

Transcription factors (TFs) are essential for the regulation of gene expression (Thomas et al., 2010); numerous transcription factors in multicellular organisms are capable of regulating the genes involved in development and functions (Marsman and Horsfield, 2012), which control crucial cellular processes like apoptosis, cell growth, and cellular differentiation. Previous studies have revealed that TFs play important roles in biparental early embryogenesis, for instance, *Foxo1*, *Foxo3*, and *Foxo4* are differently expressed during mouse oocyte maturation and preimplantation embryo development (Kuscu and Celik-Ozenci, 2015). Drosophila *STAT (STAT92E)* is present in the early embryo as a maternal product, and the expression of this gene is activated during the maternal to zygotic (MZT) process, which plays an important role in transcription of the zygotic genome at the onset of embryonic development (Tsurumi et al., 2011). As a critical transcription factor in early mammalian development, *STAT3* has likely been involved in the determination of the animal pole of the oocyte and in the establishment of the inner cell mass and trophoblast in the preimplantation embryos (Zhang et al., 2007). *USF1* is essential for oocyte maturation and early embryonic development in bovine (Datta et al., 2015). *POU5F1(OCT4)* is initially expressed at eight-cell stages, while *CDX2* is upregulated in the mouse blastocyst stages (Niakan and Eggan, 2013). More and more TFs such as *ATF3*, *EN1*, *IFI16*, *IKZF3*, *KLF3*, *NPAS3*, *NR2F2*, *RUNX1*, *SOX2*, *ZBTB20*, and *ZSCAN4* (Godini and Fallahi, 2019) genes were discovered using the new technology of single-cell sequencing.

However, due to the quantitative limitation in research material, the contribution of TFs for early uniparental embryo development which includes diploid parthenogenesis and diploid androgenesis is still largely unknown. For humans, PG or AG embryos have the potential to develop into the hydatidiform mole or ovarian teratomas (Fujita et al., 1994; Haig, 2015) respectively. The maternal *Plag1* gene is needed for mouse preimplantation embryo development. Mouse embryos lacking *Plag1* may lead to extensive gene dysregulation and prolong the time period from the one-cell to two-cell stage (Madissoon et al., 2019). *SOX2* expression is reduced in hydatidiform moles and choriocarcinomas compared with the normal placenta (Li et al., 2008). Over the last few years, it is reported that uniparental embryo stem cells are used for neural disease therapy, skin repair, and other cell therapies. Didié et al. (2013) demonstrated the pluripotency of parthenogenetic stem cells (PSCs) and suggested this unique cell type as an attractive source for tissue-engineered heart repair. Liu et al. (2017) introduced an effective and practical strategy for applying PSCs for tendon regeneration which shown that PSCs displayed fundamental properties similar to those of ESCs, including pluripotency, clonogenicity, and self-renewal capacity. International Stem Cell Corporation (ISCO) revealed that human parthenogenetic neural stem cells (ISC-hpNSC) have potential therapeutic value for patients suffering from traumatic brain injury (TBI) (Lee et al., 2019). Eckardt et al. (2007) also reported that uniparental cells of paternal origin can form adult-transplantable stem cells and can repopulate an adult organ.

In this study, we systematically studied the expression profiles of all annotated transcription factors in uniparental early embryonic cells by single-cell RNA sequencing (scRNA-seq), and we provide the first comprehensive regulatory framework of transcription factors in human uniparental and biparental early embryogenesis. Moreover, this study may pave a way to find potential risk factors for early pregnancy diseases such as hydatidiform mole and teratoma and can provide suggestions for the utilization of uniparental embryo stem cells in regenerative medicine.

## Results

### Transcription Factor Gene Expression Patterns in Biparental and Uniparental Embryos During Early Developmental Progress

**FIGURE 1:**
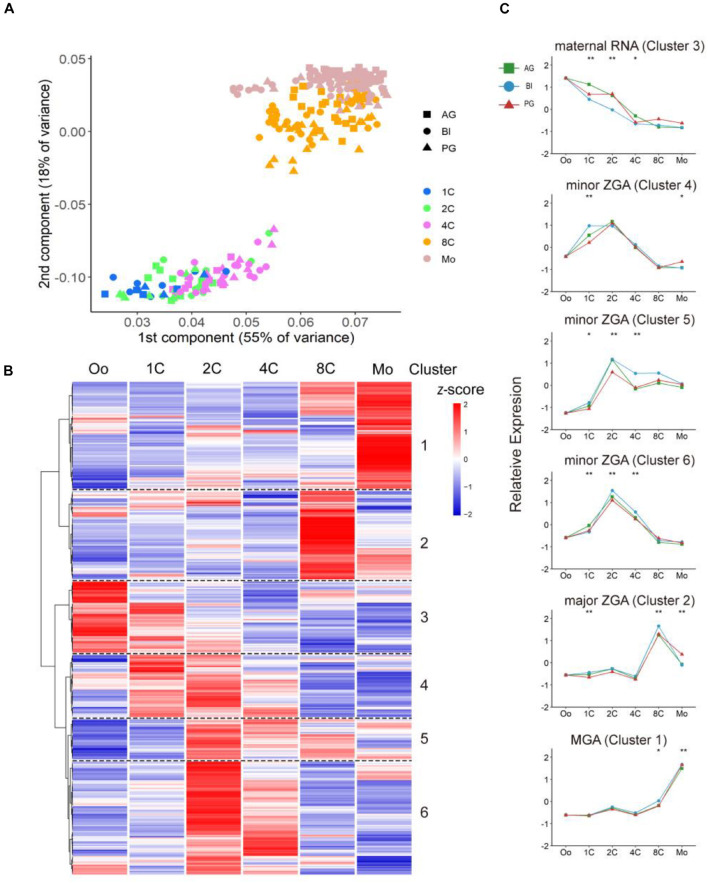
Dynamic expression pattern of TFs in biparental and uniparental embryos. **(A)** Principal component analysis of expression TFs in biparental and uniparental cells. All cells were clustered into two distinct clusters. **(B)** Clustering of expression TFs in early BI cells from oocyte to morula (uniparental cells are similar to BI cells, figures are not shown). **(C)** TFs from BI cells were classified into five modules according to their expression patterns. Line plot shows relative expression in biparental and uniparental embryos in the corresponding pattern of BI cells. *p < 0.05, **p < 0.01 (Kruskal–Wallis test).

From previous reports (Niakan and Eggan, 2013; Godini and Fallahi, 2019), TFs have important functions in initial embryonic genome activation (EGA), which involved dramatic expression changes during preimplantation development; thus, we explored the different contributions of TFs in biparental and uniparental embryonic development. AG and PG embryos possess two paternal or maternal genomes, respectively; the detailed methods of embryo construction can be found in the article of Leng et al. (2019). We used a dataset from our previous study which contains cells from the oocyte to the morula stage of AG (*n* = 89), PG (*n* = 73), and BI (*n* = 123) embryos (Leng et al., 2019; Supplementary Table 1). We used principal component analysis (PCA) to cluster both biparental and uniparental cells based on all 387 expressed TFs [median transcripts per million (TPM) ≥ 1, Supplementary Table 2]. The PCA results (Figure 1A) showed that different embryo types were randomly mixed and clustered according to their developmental stages, and the embryos at different stages were separated into two distinct clusters. The first cluster contained one-cell, two-cell, and four-cell stages. The eight-cell and morula were grouped into the second cluster, which showed that the four- to eight-cell stage was an obvious process of EGA (Braude et al., 1988; Dobson et al., 2004). Then, we profiled 123 individual cells from 31 BI embryos at 6 consecutive developmental stages, including the stages from oocyte to morula, to illustrate the dynamic changes of TFs in biparental embryos. All TFs were divided into two main groups with six clusters (Figure 1B). According to their expression pattern, we further classified them into four modules: M1: degradation of maternal mRNAs (maternal RNA) (cluster 3); M2: minor zygotic genome activation (ZGA) (clusters 4, 5, 6); M3: major ZGA (cluster 2); and M4: mid-preimplantation genome activation (MGA) (cluster 1). The features of these TF expression profiles were highly consistent with previous studies (Wang and Dey, 2006; Niakan et al., 2012).

Maternal RNA pattern TFs (cluster 3; *n* = 57, 15% of 387 expressed TFs) were rapidly degraded and steady declined after fertilization. The decreasing trend continued until the four-cell stage. Notably, this low level of expression could be maintained until the morula period. The minor ZGA pattern genes (clusters 4, 5, 6; *n* = 174, 45%) initially experienced a rapid increase after fertilization and maintained a high level of expression to the two-cell stage. For minor ZGA, *BACH1*, and *CSDE1* genes were found more highly expressed in these stages (Supplementary Figure 1A). We also found that *PIAS4* was highly expressed in the major ZGA stage (Supplementary Figure 1A), contrary to the studies in mice that *Pias4* was downregulated in the ZGA process and ESCs to 2c-like cell transformation (Yan et al., 2019). For the major ZGA pattern (*n* = 70, 18%), the peak appeared at the eight-cell stage followed by a rapid decrease of expressed TFs. We checked the expression status of some typical genes, such as *LEUTX* and DPRX, most were consistent with previous reports (Tohonen et al., 2015; Jouhilahti et al., 2016), *LEUTX* was significantly upregulated at the four-cell stage, and *DPRX* was upregulated at the eight-cell stage (Supplementary Figure 1B). MGA pattern genes (*n* = 86, 22%) initialized a rapid increase from four-cell (cluster 1) and had the maximum RNA expression at the morula stage (Figure 1C).

To investigate the correlation between TF expression pattern and development stages, Gene Ontology was performed to see the major biology function of each stage (Supplementary Figure 1C). From the dot diagram, we found that DNA-binding transcription activator activity, RNA polymerase II-specific, were enriched in all four modules. With the beginning of the ZGA process, DNA-binding transcription repressor activity and enhancer-related binding activities began to increase, indicating that TFs in this period bind mainly to enhancers to regulate the expression of adjacent genes and control ZGA process.

Next, we compared the expression patterns of biparental and uniparental embryos; although uniparental embryos showed overall a similar trajectory with biparental embryos in the whole TF expression level, some subtle differences also existed which may affect uniparental embryonic development (Figure 1C). The difference existed mainly in the degradation of maternal RNA and minor ZGA during the one-cell to four-cell stage. The degradation of material in biparental embryos showed a rapid linear decrease from the one- to four-cell stage, but both AG and PG embryos had a slow decrease from the one- to two-cell stage and a sharp decrease from the two- to four-cell stage. In the minor ZGA module (cluster 4), the gene expression of BI embryos was kept stable from the one- to two-cell stage, rapidly decreased from the two- to four-cell stage, and ZGA of uniparental embryos appeared to start at the two-cell stage.

### Differentially Expressed Transcription Factors Contribute to Uniparental Embryo Epigenetic Modification

**FIGURE 2:**
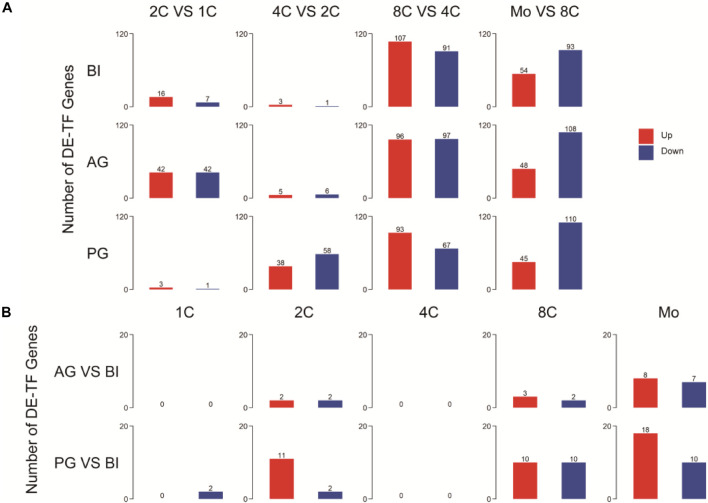
Detection and annotation of DE-TFs between and within different embryos. **(A)** DE-TFs within different types of embryos. **(B)** DE-TFs between biparental and uniparental embryos.

To further explore the contribution of TFs to uniparental embryo development, we analyzed differentially expressed TFs (DE-TFs) in consecutive developmental stages of different parental origins of embryos (p.adj ≦ 0.05 and |log2 fold change| ≧ 1) (Figure 2A). By comparing the number of expressed TFs with BI embryos, AG embryos have a more similar tendency of change with BI embryos, however, with the exception that a minor difference occurred in the one- to two-cell stage, and PG embryos had a relatively obvious difference in the two- to four-cell stage (Supplementary Figure 1D). During the one- to two-cell transition, more upregulated genes that appeared in BI embryos were related to chromatin remodeling (Supplementary Table 3) and downregulated genes that appeared in AG embryos were related to epigenetic modification (Supplementary Figure 2A).

To observe the effects of different ways of embryonic formation processes on early embryonic development, we analyzed DE-TFs among three embryo types within the same stage (p.adj≦0.05 and |log2 fold change|≧1). The list of the top five differentially expressed (upregulated and downregulated) TFs is shown in Supplementary Table 4. The number of DE-TFs in PG embryos was visibly increased in the two-cell, eight-cell, and morula stages; AG embryos showed a sensible difference in the morula stage (Figure 2B). On further analysis of these differentially expressed genes, for PG embryos, the upregulated genes at the morula stage were associated with DNA integration, and the downregulated genes were related to transmembrane receptor protein serine and threonine kinase signaling pathways (Supplementary Table 5). AG embryo-downregulated genes at the morula stage were associated with DNA methylation (Supplementary Figure 2B).

### Zf-C2H2, HMG, and MYB Are Three Dominant Transcription Factor Families in Early Embryo Development

**FIGURE 3:**
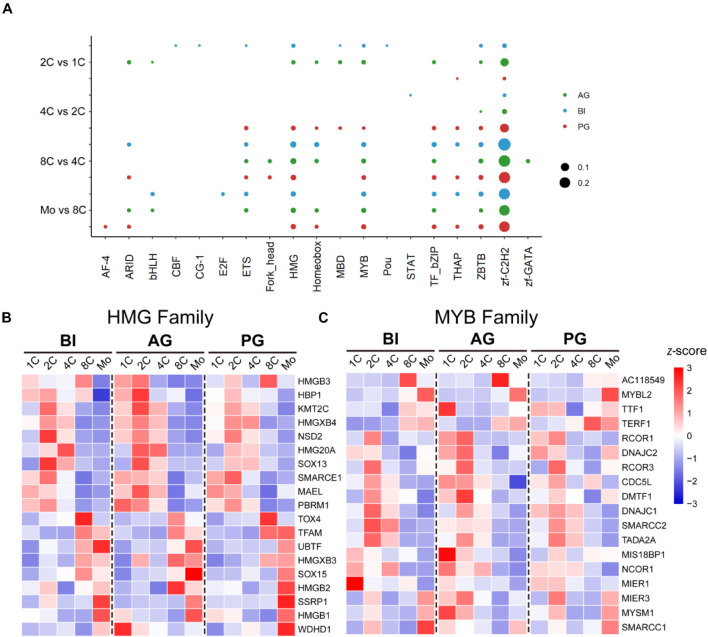
Dominant TF families in biparental and uniparental early embryogenesis based on DE-TFs in successive developmental stages. Zf-C2H2, HMG, and MYB families are the top 3 TF families. **(A)** The rate of the top 10 TF families in each transitional stage of different original embryos. **(B)** The dynamic expression of the HMG family. **(C)** The dynamic expression of the MYB family.

Transcription factors can be grouped into different families, depending on the structure of their DNA-binding domains (DBDs), and each family preferentially binds a consensus DNA sequence (Charoensawan et al., 2010). According to our data, we analyzed the distribution of TF families of DE-TFs at five stages in biparental and uniparental embryos and found that there was a similar distribution in the top three largest TF families among three different embryo types. The bubble plot (Figure 3A) showed that Zf-C2H2, HMG, and MYB TF families are three dominant TF families; Zf-C2H2 is well-known as the largest TF family as previously reported (Najafabadi et al., 2015). We focused with greater intensity on the TFs in the HMG and MYB families that contain fewer TFs and found that the TFs of the HMG family were expressed in the whole stages, but the TFs in the MYB family mainly had a higher expression in the one- and two-cell stages; the expression patterns were similar between BI, AG, and PG embryos (Figures 3B,C).

### Network Construction and Analysis of Transcription Factors and Their Target Genes

As TFs initialize gene transcription through binding to the gene promoter, we focused intensively on TFs with known transcription factor binding sites (TFBSs) to explore the gene co-expression profile during early embryo development by constructing their co-expression network with potential target genes (Mandevar et al., 2012; Lelieveld et al., 2016).

**FIGURE 4:**
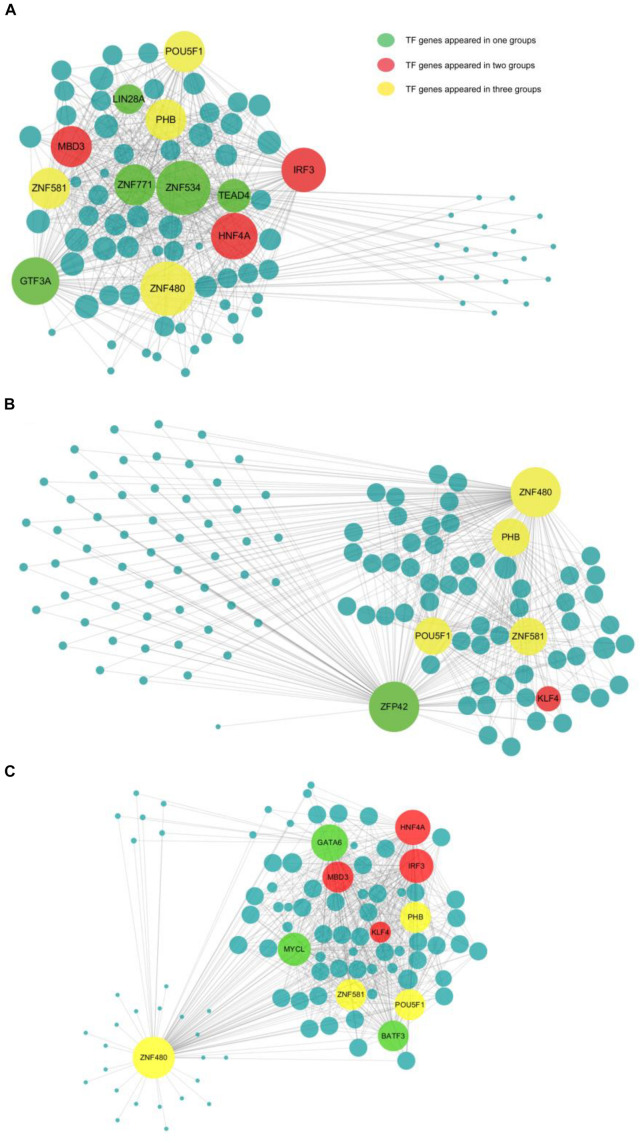
The network information involved in biparental and uniparental embryos. The size of a node is related to the number of genes that interact with it. The more genes there are, the bigger the node. The line between two nodes shows the degree of interaction. The more correlation there is, the shorter the line. Yellow is used to show common TFs in all three groups; red is used to show common TFs in two groups; and green is used to show special TF in one group. **(A–C)** The network information involved in AG, PG, and BI embryos, showing the 12, 6, and 11 most important TFs together with their co-expressed target genes, respectively.

There were 11, 12, and 6 TFs found in the main regulatory network in BI, AG, and PG embryos, respectively (Figure 4 and Table 1). *POU5F1*, *PHB*, *ZNF581*, and *ZNF480* were the four shared hub TFs in all types of embryos. AG and BI embryos shared *HNF4A*, *IRF3*, and *MBD3* three hub TFs, whereas *KLF4* was the only shared hub TFs in PG and BI embryos. Besides shared hub TFs, the dominant hub TFs in each embryo were also identified; *LIN28A*, *ZNF771*, *ZNF534*, *TEAD4*, and *GTE3A* belonged to AG embryos. *ZFP42* only appeared in the main regulatory network in PG embryos, while *GATA6*, *MYCL*, and *BATF3* were only found in BI embryos. To further analyze the expression of TFs in three cell types, we found that almost all hub TFs showed an up-trend and had an extremely high expression in the morula stage; *MBD3* was highly expressed in AG and BI embryos; *KLF4* was highly expressed in BI and PG embryos; *ZNF534*, *GTF3A*, and *LIN28A* were highly expressed in AG embryos; *GATA6* and *MYCL* were highly expressed in BI embryos; and *ZFP42* was higher in PG embryos (Supplementary Figure 3A). These hub genes are relatively specific to embryo types and are consistent with the results of co-expression regulatory network analysis.

**TABLE 1:** Detailed information of detected hub TFs.

Symbol	Group	Chromosome	TF family
ZNF480	AG,BI,PG	19	Zf-C2H2
ZNF581	AG,BI,PG	19	Zf-C2H2
PHB	AG,BI,PG	17	Others
POU5F1	AG,BI,PG	6	Pou
HNF4A	AG,BI	20	RXR-like
IRF3	AG,BI	19	IRF
MBD3	AG,BI	19	MBD
KLF4	BI,PG	9	Zf-C2H2
ZNF534	AG	19	Zf-C2H2
GTF3A	AG	13	Zf-C2H2
ZNF771	AG	16	Zf-C2H2
TEAD4	AG	12	TEA
LIN28A	AG	1	CSD
GATA6	BI	18	Zf-GATA
MYCL	BI	1	bHLH
BATF3	BI	1	TF_bZIP
ZFP42	PG	4	Zf-C2H2

To learn more about the function of these hub TFs, we performed gene annotations for target genes of hub TFs in uniparental and biparental embryos (Supplementary Figure 3B). The dominant biological processes were enriched in stem cell population maintenance and differentiation across the three different types of embryos. As confirmed in previous studies *POU5F1*, a critical TF for early embryo development and embryonic stem cell pluripotency has been confirmed in previous studies (Niakan and Eggan, 2013). By analyzing the target gene expression of *POU5F1* from the four- to eight-cell stages, it was found that *MYC*, *YY1*, *SOX2*, *SALL4*, and *KLF4* genes were significantly upregulated, while *SMAD1* gene expression was significantly decreased (Supplementary Figure 3C). Among these target genes, *ZFP42*, also known as *REX1*, which was found to play an important role in the development of inner cell mass (ICM) in mice (Toyooka et al., 2008), was dominantly presented in the PG embryo regulatory network.

### Functional Enrichment Analysis of Hub Transcription Factors Using the Public Database

**FIGURE 5:**
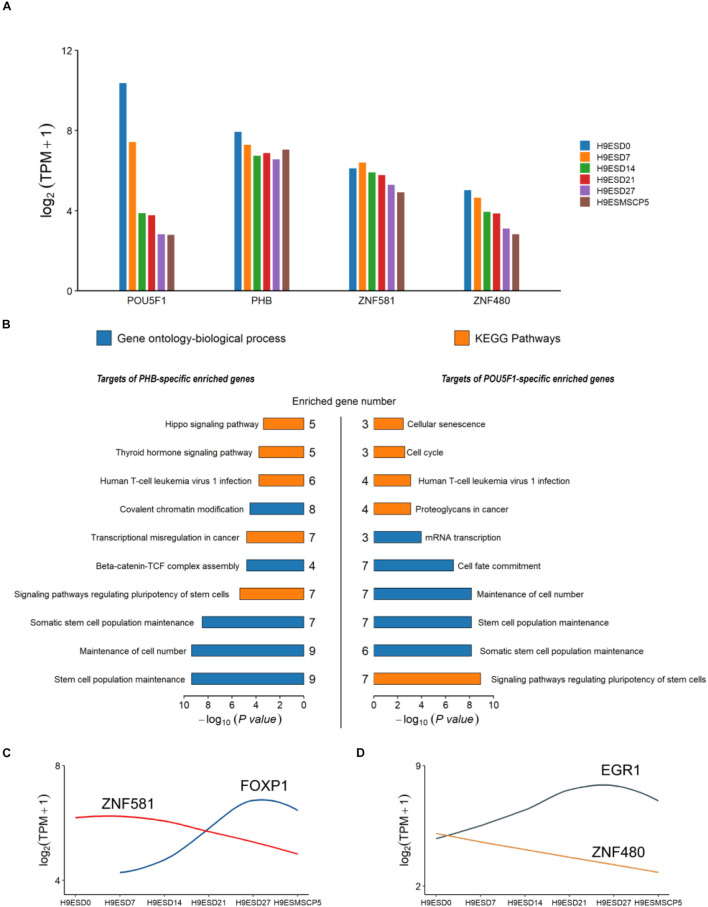
The expression and annotation of four common TFs which appeared in all three types of embryo. **(A)** Expression of POU5F1, PHB, ZBF581, and ZNF480 from the human embryonic stem cell (hESC) to the induced mesenchymal stem cell (MSC). **(B)** Gene Ontology (GO) and KEGG pathways enriched for target genes of PHB and POU5F1. The *p*-values were calculated using the Benjamini-corrected test. **(C,D)** The dynamic expression for ZNF581 and ZNF480 and their target genes.

Human embryonic stem cell derived-mesenchymal stem cells (hESC-MSC) are regarded as an appropriate and valuable cell model for exploring the function of TFs in embryo development (Zou et al., 2013). To investigate the role of *POU5F1*, *PHB*, *ZNF581*, and *ZNF480*, the hESC-MSC differentiation cell model was generated. RNA-seq was done for hESC at four time points D7, D14, D21, and D27 during the differentiation process (CNP0000771). It was found that all the four genes had a high expression in hESC and rapidly decreased during the hESC-MSC differentiation process. *PHB* kept a high expression level and even increased a little in the MSC stage, *ZNF581* showed a slight decrease, and the *ZNF480* gene expression decreased over time (Figure 5A). From the GO and KEGG results, we could find that the targets of POU5F1 and PHB were both enriched in maintenance of the stem cell population and cell number (Figure 5B). The *SOX2*, *STAT3*, *NANOG*, and *SALL4* genes which were found to play important roles in different stages of early embryo development stages were also enriched in these two terms (Supplementary Tables 6, 7). The annotated target genes of *ZNF581* and *ZNF480* were fewer than *POU5F1* and *PHB*. It was noteworthy to find that *FOXP1* was one of the *ZNF581* target genes which were faintly expressed in hESCs, and its expression was upregulated during iPSC-MSC differentiation and its expression was kept relatively high at the mature MSC stage. This suggests that *FOXP1* may play an important role to trigger human ESC to differentiate to MSC; similar results were also obtained from the work done by Gabut et al. (2011). *ZNF581* binding to the *FOXP1* gene domain may affect the hESC differentiation process (Figure 5C); however, conclusive proof will require additional experiments. EGR1 regulated by the *ZNF480* gene has a similar expression tendency with *FOXP1* and its potential function in promoting cell proliferation and differentiation (Figure 5D; Tsugata et al., 2018).

### Methylation Status in the Promoter Region of Transcription Factors in Three Types of Embryos

**FIGURE 6:**
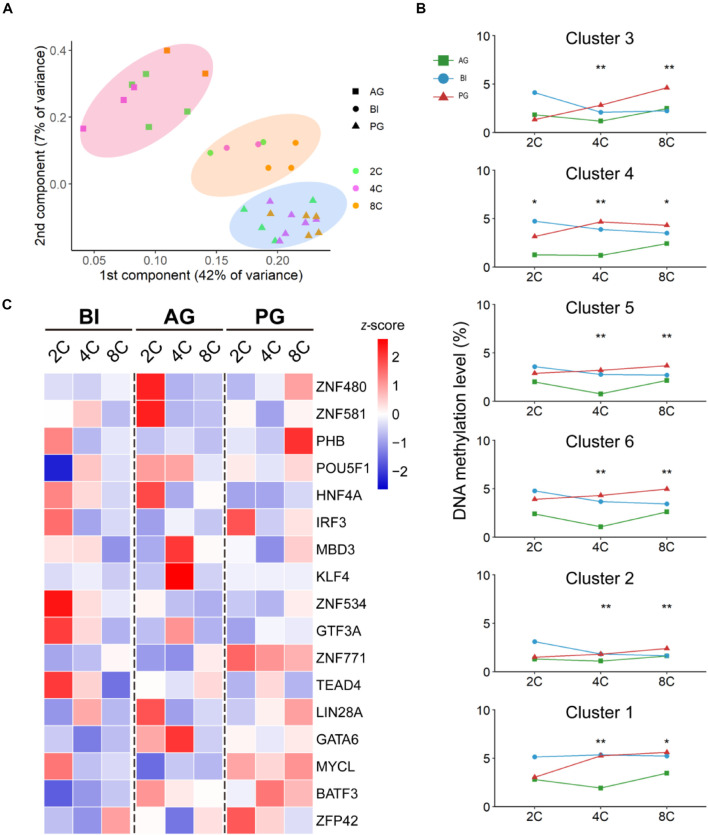
The promoter CPG-methylation state in biparental and uniparental cells. **(A)** PCA of promoter methylation of all detected TFs. **(B)** Mean promoter methylation tendency of each development module, corresponding to clusters in BI embryos. *p < 0.05, **p < 0.01 (Kruskal–Wallis test). **(C)** Promoter methylation (mean ± SE) of 17 hub TFs.

An increasing body of evidence shows that promoter methylation is one of the common ways to impact gene expression (Héberlé and Bardet, 2019; Zhou et al., 2019). However, how the methylation state in the promoter region of TFs affects the expression of target genes in early embryo development is still unclear. Using the single-embryo whole-genome bisulfite-seq (WGBS) strategy, we analyzed all the methylation levels of TF promoters in BI, AG, and PG cells from the two- to eight-cell stage, in a total of 31 individual cells (Supplementary Table 8). We performed PCA on all detected 1,599 TFs’ promoter regions, and the results showed that all cells were grouped by embryo types, which differed from the results obtained by profiling their gene expression (Figure 6A). It indicated that the methylation difference of TF genes has a significant correlation with embryo types. Subsequently, we calculated the mean methylation level of each of the mentioned expression patterns, corresponding to each cluster (Figure 6B); dramatic differences appeared in uniparental and biparental embryos at the four-cell and eight-cell stages; AG embryos had the lowest methylation level at the four-cell and eight-cell stages; and PG embryos demonstrated the highest methylation at the eight-cell stage. We then focused on the hub TFs mentioned above. Most TFs maintained a low methylation degree except *POU5F1*, *HNF4A*, and *ZNF534* (Figure 6C). We then searched all target genes of *POU5F1*, *HNF4A*, and *ZNF534* to explore the methylation and expression correlation and found that correlation is not obvious between promoter region methylation and the target gene expression level regulated by TFs during early embryo development (figures were not shown here).

## Discussion

TFs’ expression profiles are critically important in early embryo development. In this study, based on the single-cell RNA-seq data regarding biparental and uniparental embryos from zygote to morula stages, we categorized expressed TFs in both uniparental and biparental embryos most likely as maternal RNA, minor ZGA, major ZGA, and MGA patterns. We further explored the contribution of TFs to the biological processes and key events in these five early stages in both uniparental and biparental embryos. In general, the expression patterns were very similar within the uniparental and biparental early embryogenic processes, which was highly consistent with previous studies (Wang and Dey, 2006; Niakan et al., 2012). *BACH1* and *CSDE1* genes were found with higher expression in the minor ZGA stage, *PIAS4* was highly expressed in the major ZGA stage, *LEUTX* was significantly upregulated at the four-cell stage, and *DPRX* was upregulated at the eight-cell stage; these genes are especially expressed in a certain period and acted as crucial genes for stage-related events, which also have the potential to act as marker genes to evaluate embryonic development status during *in vitro* embryo culture and are of significant value for the ongoing efforts to improve *in vitro* fertilization technology.

Transcription factors belong to different TF families; the results of this study show that Zf-C2H2, HMG, and MYB were three dominantly expressed TF families in early embryo development. Zf-C2H2 is essential for transcriptional regulation and development/differentiation of organs/tissues in the early embryonic stage (Mackeh et al., 2018). The HMG family is the earliest detected TF family expressed during embryonic development, and it is responsible for regulating progenitor cell specification and the terminal differentiation of multiple cell types in a wide variety of lineages (Sarkar and Hochedlinger, 2013). Current understanding is that the MYB family has a close correlation with tumorigenesis (Ramsay and Gonda, 2008); it was enlightening to find that the MYB family is also highly expressed in the one- and two-cell stage of three types of embryos and possibly has more responsibility for embryonic cell proliferation.

To explore the role of the four shared hub TFs in early embryo development, we checked the expression of these four genes in hESC at several time points during the process of hESC differentiation toward hESC-MSCs; we found that their expression tendency and functions in embryonic development and differentiation are very similar, highly expressed in hESC, and less expressed in the differentiation process. Most of their targeted genes were involved in maintaining cell pluripotency and cell fate determination. *POU5F1* is a well-known marker for maintaining and regulating embryonic cell pluripotency, and PHB can effectively maintain and retain the pluripotent state of hESC (Shi and Jin, 2010; Zhu et al., 2020). The role of *ZNF581* and *ZNF480* requires additional investigation (Yi et al., 2004; Walsh et al., 2017). In our study, it was predicted to work as inhibitors for *FOXP1* and *EGR1* to promote stem cell proliferation and differentiation separately. Further studies are needed to uncover their functions.

The DNA methylation status of all promoter regions of TFs was analyzed to investigate whether it is a potential influencing factor for TFs in the regulations of embryo development activities. An overall observation found that all embryos were grouped by parental origin rather than developmental stages; this result showed that the process of embryo construction has a great effect on parental genome methylation. For the hub TFs, although there existed slight differences among AG, PG, and BI embryos, the transcript expression level had no significant difference, which may infer that there are some other important factors that may act on TFs to affect hub gene expression.

In summary, this is a relatively systematic work describing the dynamic changes of TFs for uniparental and biparental embryogenesis by single-cell sequencing data. It provides a baseline to understand the mechanism of TF regulation in early biparental and uniparental embryo development. The exploration of shared and unique hub TFs for biparental and uniparental embryo development also provides some clues for the utilization of uniparental embryonic stem cells in regenerative medicine and stem cell therapy areas.

## Materials and Methods

### Data Preparation

All RNA-seq data and WGBS data of early embryonic development came from a freely available data set deposited by our previous study at the Gene Expression Omnibus (GEO) under accession number GSE133856^1^. This data set contains stages from oocyte to morula in androgenesis (AG, *n* = 89), parthenogenesis (PG, *n* = 73), and biparental (BI, *n* = 123) embryos. We had a total of 285 available cells from oocyte to morula and then identified 387 expressed TFs (median TPM ≧1) based on animal TFDB (Zhang et al., 2012) human TFs for subsequent analysis.

### Transcription Factor Gene Expression Profile in Biparental Embryos

We clustered all 387 expressed TFs in 123 individual cells from 31 BI human early embryos at 6 consecutive developmental stages by using the pheatmap package, including the stages from oocyte to morula. The TPM median of all cells from the same stage is used to characterize the expression level at this stage. The average expression of all TFs in the corresponding cluster represents the expression level of each stage. We made line plots of the relative expression level of TFs in each cluster of BI embryos and annotated each expression pattern in terms of Gene Ontology: Biological Process using clusterProfiler package (Yu et al., 2012).

### The Effect of Parents on Early Embryo Development and Dominant Transcription Factor Families in Early Embryo Development

Principal component analysis (PCA) was performed to cluster biparental and uniparental embryos. Then, we made line plots of the relative expression levels of TFs for biparental and uniparental embryos according to the expression patterns of biparental embryos. Furthermore, the DESeq2 (Love et al., 2014) package was used to detect differential expression TFs (DE-TFs) in five consecutive developmental stages from one-cell to morula. Then, we counted the top 10 largest TF families of DE-TFs in consecutive developmental stages of different embryos with reference to the ChEA database (Lachmann et al., 2010).

### Network Construction and Analysis

All DE-TFs and their target genes were applied to construct the co-expressed network using the information of the ChEA database (Lachmann et al., 2010). In this process, network construction was achieved by the WGCNA package (Langfelder and Horvath, 2008) and the visualization of the core regulatory network came true by Cytoscape software (Shannon et al., 2003). Moreover, genes that interacted with hub TFs were executed by Gene Ontology annotation.

### Validation of Common Transcription Factors Which Appeared in All Three Types of Embryo in H9ES

We collected partial RNA-seq data (CNSA accession number: CNP0000771) of the paper published by Luo et al. (2020), which was during iMSC (induced mesenchymal stromal cell) derivation from H9ES cells on Days 0, 7, 14, 21, 27 (passage 0, P0), and P5, to view the expression changes of some key transcription factors during this induced differentiation^2^. Moreover, the target genes of hub common TFs were executed from Gene Ontology annotation and Kyoto Encyclopedia of Genes and Genomes (KEGG) for pathway enrichment analysis.

### Methylation Analysis of Transcription Factors’ Promoter Region

All genes’ promoter region methylation levels were calculated using Perl scripts. The median methylation rate was used to represent the methylation level of each stage in embryos. In addition, the whole genome’s methylation data were uploaded in the CNGB database (see text footnote 2), which are available for further analysis.

## Data Availability Statement

The datasets presented in this study can be found in online repositories. The names of the repository/repositories and accession number(s) can be found below: GEO accession number: GSE133856, CNSA accession number: CNP0000771.

## Ethics Statement

The studies involving human participants were reviewed and approved by the Reproductive and Genetic Hospital of CITIC-XIANGYA (Research license LL-SC-SG-2013-012). The patients/participants provided their written informed consent to participate in this study.

## Author Contributions

JH, GeL, and FX conceived and designed this study. CZ, CL, and JW analyzed the data. FF, GuL, LLe, and LLi performed the experiments. DZ, XL, YL, and LB supervised the project. CZ, CL, LY, and DJ designed and wrote the manuscript. All authors contributed to the article and approved the submitted version.

## Conflict of Interest

CZ, CL, LY, DJ, JW, FF, GuL, YL, LB, JH, and FX were employed by BGI group. The remaining authors declare that the research was conducted in the absence of any commercial or financial relationships that could be construed as a potential conflict of interest.

## Publisher’s Note

All claims expressed in this article are solely those of the authors and do not necessarily represent those of their affiliated organizations, or those of the publisher, the editors and the reviewers. Any product that may be evaluated in this article, or claim that may be made by its manufacturer, is not guaranteed or endorsed by the publisher.

## Footnotes

1
https://www.ncbi.nlm.nih.gov/geo/query/acc.cgi?acc=GSE133856

2
https://db.cngb.org/search/project/CNP0000771
