# Supplementary material for: The Dynamic Changes of Transcription Factors During the Development Processes of Human Biparental and Uniparental Embryos
Source: Front Cell Dev Biol. 2021 Sep 17;9:709498. doi: 10.3389/fcell.2021.709498 (PMC8484909; doi:10.3389/fcell.2021.709498)
Supplement: Supplementary file 1 [file Data_Sheet_1.pdf]

Supplementary Figures

Figure S1

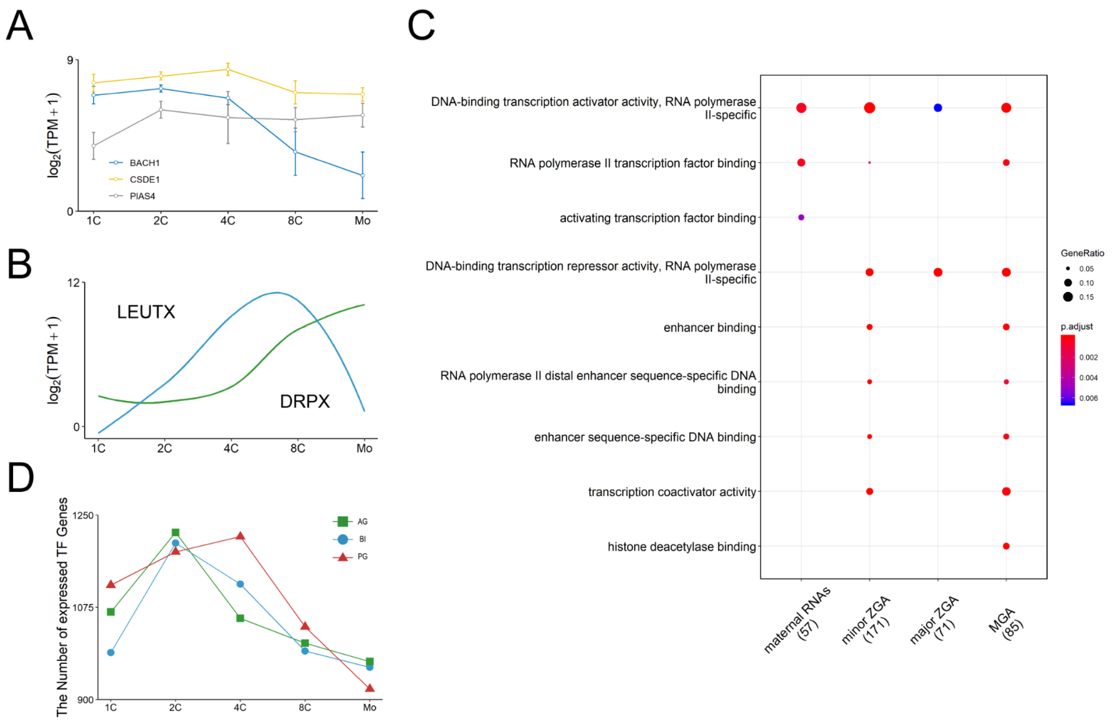

Figure S1. Dynamic expression and annotation of TFs.

Related to Figure 1.

(A) The dynamic expression of BACH1, CSDE1, and PIAS4.

(B) The dynamic expression of LEUTX and DRPX.

(C) Gene annotation of corresponding modules in BI embryos.

(D) All detected TFs in the developmental stages of biparental and uniparental embryos.

Figure S2

A

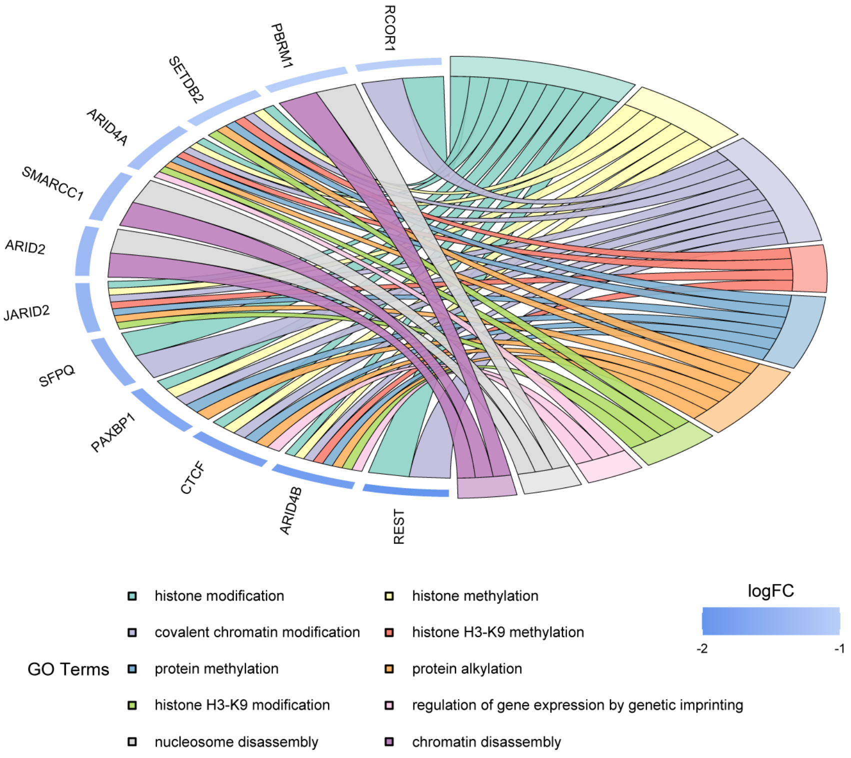
RCOR1
PBRM1
SETDB2
ARID4A
SMARCC1
ARID2
JARID2
SFPQ
PAXBP1
CTCF
ARID4B
REST
GO Terms
histone modification
covalent chromatin modification
protein methylation
histone H3-K9 modification
nucleosome disassembly
histone methylation
histone H3-K9 methylation
protein alkylation
regulation of gene expression by genetic imprinting
chromatin disassembly
logFC
-2
-1

B

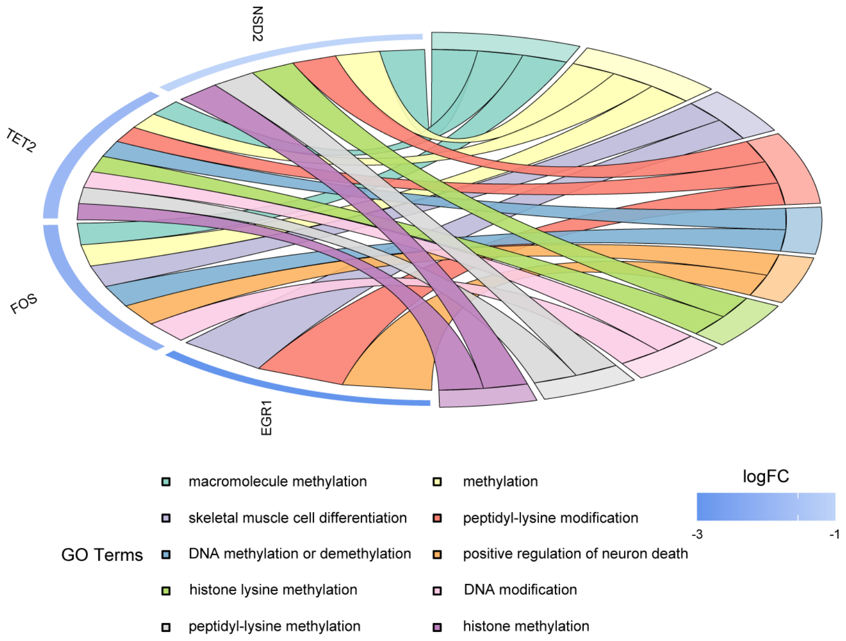
NSD2
TET2
FOS
EGR1
GO Terms
macromolecule methylation
skeletal muscle cell differentiation
DNA methylation or demethylation
histone lysine methylation
peptidyl-lysine methylation
methylation
peptidyl-lysine modification
positive regulation of neuron death
DNA modification
histone methylation
logFC
-3
-1

Figure S2. Gene annotation of differentially expressed TFs.

Related to Figure 2.

(A) GO terms of down-regulated TFs from one- to two-cell stage in AG embryos.

(B) GO terms of down-regulated TFs at morula stage comparing AG embryos to BI embryos.

Figure S3

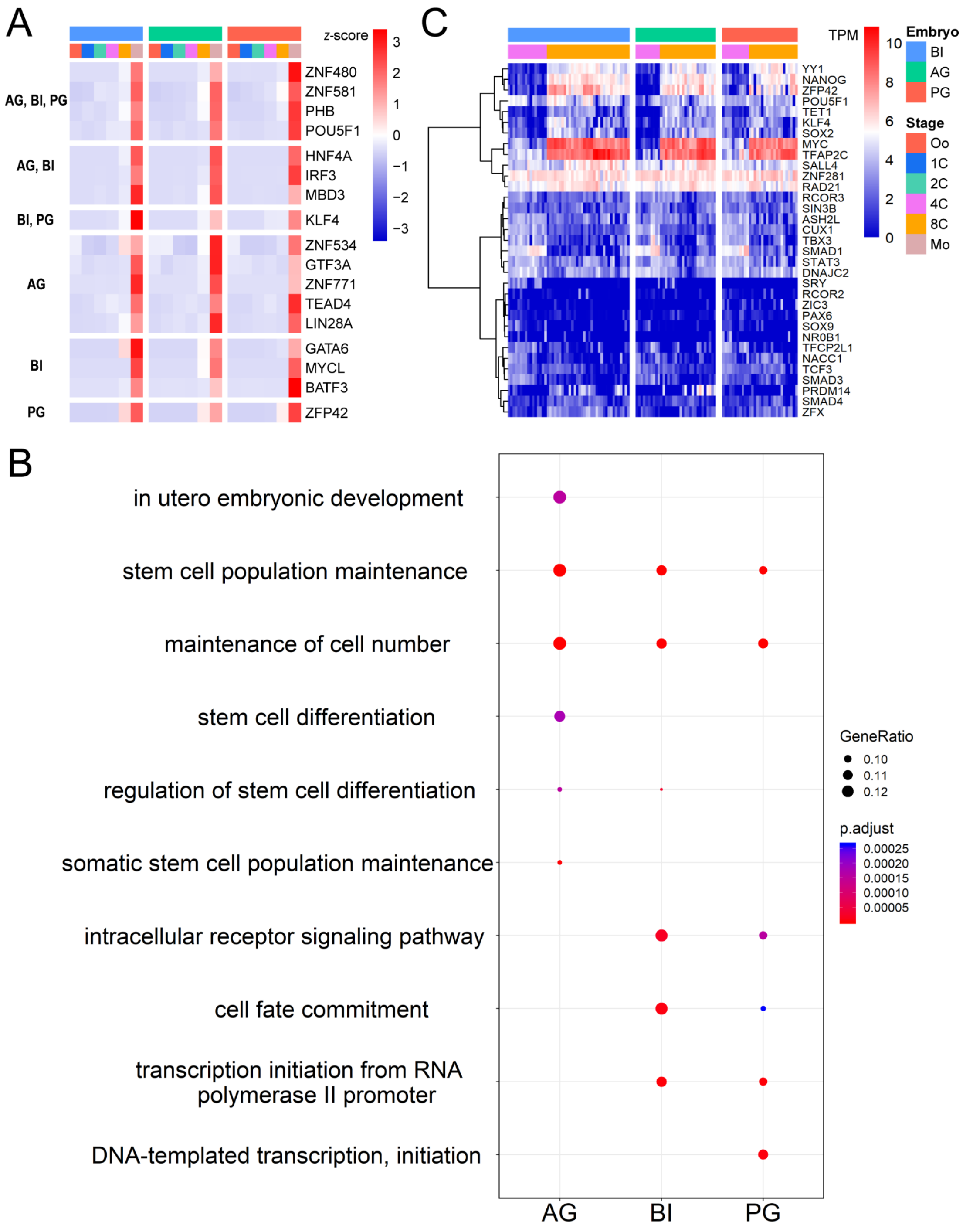

A
z-score
3
2
1
0
−1
−2
−3
AG, BI, PG
AG, BI
BI, PG
AG
BI
PG
ZNF480
ZNF581
PHB
POU5F1
HNF4A
IRF3
MBD3
KLF4
ZNF534
GTF3A
ZNF771
TEAD4
LIN28A
GATA6
MYCL
BATF3
ZFP42
C
TPM
10
8
6
4
2
0
Embryo
BI
AG
PG
Stage
Oo
1C
2C
4C
8C
Mo
YY1
NANOG
ZFP42
POU5F1
TET1
KLF4
SOX2
MYC
TFAP2C
SALL4
ZNF281
RAD21
RCOR3
SIN3B
ASH2L
CUX1
TBX3
SMAD1
STAT3
DNAJC2
SRY
RCOR2
ZIC3
PAX6
SOX9
NR0B1
TFCP2L1
NACC1
TCF3
SMAD3
PRDM14
SMAD4
ZFX
B
in utero embryonic development
stem cell population maintenance
maintenance of cell number
stem cell differentiation
regulation of stem cell differentiation
somatic stem cell population maintenance
intracellular receptor signaling pathway
cell fate commitment
transcription initiation from RNA polymerase II promoter
DNA-templated transcription, initiation
AG
BI
PG
GeneRatio
0.10
0.11
0.12
p.adjust
0.00025
0.00020
0.00015
0.00010
0.00005

Figure S3. Dynamic expression and annotation of genes.

Related to Figure 4.

(A) Heatmap of dynamic expression of hub TFs in biparental and uniparental embryos.

(B) Annotation of target genes of hub TFs in different original embryos.

(C) The target gene expression of POU5F1.
